# Downregulated Regucalcin Expression Induces a Cancer-like Phenotype in Non-Neoplastic Prostate Cells and Augments the Aggressiveness of Prostate Cancer Cells: Interplay with the G Protein-Coupled Oestrogen Receptor?

**DOI:** 10.3390/cancers16233932

**Published:** 2024-11-24

**Authors:** Lara R. S. Fonseca, Ricardo J. P. Carreira, Mariana Feijó, José E.B. Cavaco, Henrique J. Cardoso, Cátia V. Vaz, Marília I. Figueira, Sílvia Socorro

**Affiliations:** CICS-UBI—Health Sciences Research Centre, University of Beira Interior, 6201-001 Covilhã, Portugal; lara.fonseca@ubi.pt (L.R.S.F.); ricardo.carreira@ubi.pt (R.J.P.C.); mariana@fcsaude.ubi.pt (M.F.); jcavaco@fcsaude.ubi.pt (J.E.B.C.); henrique10mc@gmail.com (H.J.C.); d793@fcsaude.ubi.pt (C.V.V.); marilia.figueira@fcsaude.ubi.pt (M.I.F.)

**Keywords:** prostate cancer, regucalcin, survival, metabolism, metastisation, G-protein-coupled oestrogen receptor

## Abstract

Background/Objectives: Regucalcin (RGN) is a calcium-binding protein and an oestrogen target gene, which has been shown to play essential roles beyond calcium homeostasis. Decreased RGN expression was identified in several cancers, including prostate cancer (PCa). However, it is unknown if the loss of RGN is a cause or a consequence of malignancy. Also, it needs confirmation if RGN oestrogenic regulation occurs through the G-protein-coupled oestrogen receptor (GPER). This study investigates how *RGN* knockdown affects prostate cell fate and metabolism and highlights the GPER/RGN interplay in PCa. Methods: Bioinformatic analysis assessed the relationship between *RGN* expression levels and patients’ outcomes. *RGN* knockdown (siRNA) was performed in non-neoplastic prostate and castration-resistant PCa. Wild-type and *RGN* knockdown PCa cells were treated with the GPER agonist G1. Viability (MTT), proliferation (Ki-67 immunocytochemistry), apoptosis (caspase-3-like activity) and migration (Transwell assays) were evaluated. Spectrophotometric analysis was used to determine glucose consumption, lactate production and lactate dehydrogenase activity. Lipid content was assessed using the Oil Red assay. Results/conclusions: Bioinformatic analysis showed that the loss of *RGN* correlates with the development of metastatic PCa and poor survival outcomes. *RGN* knockdown induced a cancer-like phenotype in PNT1A cells, indicated by increased cell viability and proliferation and reduced apoptosis. In DU145 PCa cells, *RGN* knockdown augmented migration and enhanced the glycolytic profile, which indicates increased aggressiveness, in line with patients’ data. GPER activation modulated RGN expression in PCa cells and *RGN* knockdown in DU145 cells influenced GPER actions, which highlighted an interplay between these molecular players with relevance for their potential use as biomarkers or therapeutic targets.

## Simple Summary

Regucalcin (RGN) is a calcium-binding protein and an oestrogen target gene. Decreased RGN expression was identified in prostate cancer (PCa). However, it is unknown if the loss of RGN is a cause or a consequence of malignancy. Also, it needs confirmation if RGN oestrogenic regulation occurs through the G-protein-coupled oestrogen receptor (GPER). This study investigates how *RGN* knockdown affects prostate cell fate and metabolism and highlights the GPER/RGN interplay in PCa. Bioinformatic analysis of patients’ data demonstrated that the loss of *RGN* correlates with the development of metastatic PCa and poor survival outcomes. *RGN* knockdown in an *in vitro* approach induced a cancer-like phenotype in non-neoplastic prostate cells and increased the aggressiveness features of PCa cells. GPER activation modulated RGN expression in PCa cells and *RGN* knockdown influenced GPER actions, which highlighted an interplay between these molecular players with relevance for their potential use as biomarkers or therapeutic targets.

## 1. Introduction

Prostate cancer (PCa) is the 2^nd^ most common cancer in men and the fifth leading cause of cancer death worldwide [1,2]. According to Globocan 2022, Europe presents the highest incidence of PCa and the second highest mortality rate [3,4]. PCa-associated deaths are a consequence of the unknown aetiology of the disease and mechanisms that drive the progression of the disease to the more aggressive castration-resistant stages for which therapeutic options are limited and not very effective [5,6]. Epidemiologic studies over the years have tried to elucidate the different risk factors that contribute to PCa onset and progression. However, ageing is still considered the primary cause related to the development of this human neoplasia. PCa is more common in men above 50 years of age, and a peak of incidence occurs in men of approximately 70 years old [7,8,9,10]. It is accepted that ageing alters the prostate microenvironment, inducing several cellular alterations, such as increased oxidative stress and DNA damage, with the disruption of the gene expression landscape [11].

Regucalcin (RGN) is a multifunctional Ca^2+^-binding protein discovered in 1978 [12], which plays a role in maintaining intracellular Ca^2+^ levels and presents a distinctive characteristic of a significantly diminished expression with ageing [13,14,15,16,17]. This biological pattern identified in several tissues [13,14,15,16,17] has stimulated the scientific community to investigate RGN’s functions beyond Ca^2+^ homeostasis [18,19]. Indeed, RGN has been shown to regulate multiple signalling pathways related to the hallmarks of cancer [18]. Several studies performed in human cancer cell lines and rodents have demonstrated that RGN plays a significant role in controlling cell fate, namely in suppressing cell proliferation, migration and invasion [20,21,22,23,24,25,26,27,28]. RGN also has been indicated as an important protein in the control of apoptotic cell death and energy metabolism [13,20,21,22,23,24,25,29,30,31,32,33,34,35].

Previous studies of our research group and others started disclosing the role of RGN in prostate tissue homeostasis and carcinogenesis. Immunohistochemistry analysis of human PCa cases revealed that RGN expression decreases with the cellular differentiation of prostate adenocarcinoma [36]. It was also shown that patients with reduced RGN expression levels present shorter recurrence-free, progression-free and overall survival [37,38,39]. *In vitro* and *in vivo* studies showed that RGN suppresses PCa cell proliferation [37,38,39], migration and invasion [38,39,40]. Interestingly, it was found in rat prostate that RGN regulation of apoptosis is age-dependent, counteracting age-associated prostate growth and resistance to apoptosis [13,32]. Moreover, RGN’s action in suppressing glycolytic metabolism and regulating lipid metabolism has also been reported [29,33,34,35]. Although the available literature establishes a relationship between loss of RGN and prostate carcinogenesis, it is unknown if its decreased expression is a cause or a consequence of PCa and how the loss of this protein contributes to the progression of the disease. Furthermore, most studies have focused on RGN’s function in PCa cells, being warranted to investigate what happens in non-neoplastic prostate cells upon downregulation of RGN expression.

Another relevant research focus has explored the factors and mechanisms that govern and control RGN expression in distinct tissues and cells [18,41,42,43,44,45]. Among a panoply of agents, steroid hormones were identified as important regulators in maintaining RGN expression levels and RGN was pointed out as an androgen- and oestrogen-target gene [32,36,46]. The oestrogenic regulation of RGN expression in the prostate of rats and calves was described [46,47]. Interestingly, in human breast cancer MCF-7 cells, 17β-oestradiol’s actions in regulating RGN expression levels were suggested to be mediated by the membrane-bound G-protein-coupled oestrogen receptor (GPER) [36]. This receptor mediates the rapid non-genomic responses of oestrogens displaying anti-tumorigenic activity in several types of cancer [48,49]. In PCa, GPER has been linked to the regulation of cell growth, invasion and migration through the intersection of numerous oncogenic pathways [48,50]. The available data raise the question of whether GPER’s anti-tumorigenic actions can result from a relationship with RGN.

The present study aims to disclose how *RGN* knockdown affects prostate cell fate and metabolism. For this purpose, *RGN* gene expression was knocked down in human non-neoplastic prostate cells by small interfering RNA (siRNA) compared to castration-resistant PCa cells. *In silico* analysis of RGN expression in patients’ datasets and its relationship with patients’ data and clinical outcomes complemented the analysis. Moreover, this work investigated the putative interplay between GPER and RGN and its importance in modulating different cancer hallmarks.

## 2. Materials and Methods

### 2.1. In Silico Analysis

*In silico* bioinformatic analysis was performed using the CancerTool resource (http://genomics.cicbiogune.es/CANCERTOOL/index.html, accessed on 30 May 2024) [51]. This web-based platform uses public transcriptomics datasets from different cancer types, overcoming limitations found in other tools. Expression analysis of genes of interest is provided rapidly and comprehensively in well-annotated cancer datasets and is accompanied by customised reports. CancerTool also allows gene-to-gene correlations to be performed in multiple datasets. The CancerTool resource was used to analyse *RGN* mRNA expression correlated with PCa patients’ clinicopathological data and associated with cell migration and invasion (gene-to-gene correlations), using the Glinsky et al. [52], Grasso et al. [53], Lapointe et al. [54], Taylor et al. [55], TCGA [56], Tomlins et al. [57] and Varambally et al. [58] datasets (Table 1).

### 2.2. Reagents

All chemical reagents, culture medium and antibodies were acquired from Sigma-Aldrich (St. Louis, MO, USA) unless stated otherwise. The solvent used in the preparation of all solutions was ultra-pure water.

### 2.3. Cells and Treatments

Human non-neoplastic (PNT1A) and neoplastic (LNCaP, DU145 and PC3) cell lines were purchased from the European Collection of Cell Cultures (ECACC, Salisbury, UK) and the American Type Culture Collection (ATCC, Manassas, VA, USA), respectively. PNT1A cells are widely used to investigate the response of non-neoplastic prostate epithelial cells to different experimental environments [59,60]. LNCaP cells were isolated from lymph node metastases and express the androgen receptor (AR), being responsive to androgens’ actions [61,62]. DU145 cells were isolated from brain metastasis of undifferentiated grade IV prostate adenocarcinomas [63]. The epithelial PC3 cell line originated from bone metastasis of a grade IV prostate adenocarcinoma [64]. Both DU145 and PC3 cells are AR-null and widely used as a model of castration-resistant PCa, though DU145 cells represent a more aggressive stage of disease [62,63,64].

All cell lines were maintained in RPMI 1640 phenol red culture medium (R6504), supplemented with 10% fetal bovine serum (FBS, F7524) and 1% penicillin/streptomycin (P/S, A5955) at pH 7.4, in an incubator equilibrated with 5% CO_2_ at 37 °C.

For transfection and *RGN* knockdown experiments, PNT1A and DU145 cells were seeded in RPMI 1640 phenol red culture medium (10% FBS) and RPMI 1640 culture medium without phenol red (R8755) supplemented with 5% charcoal-stripped FBS (F6765), respectively. PNT1A and DU145 cells (50% confluence) were transfected with Lipofectamine RNAiMAX and 10 nM of siRNA targeting *RGN* (si-*RGN*, s17374, Ambion, Carlsbad, CA, USA) or scrambled siRNA (si-Scr, sc-37007, Santa Cruz Biotechnology, Santa Cruz, CA, USA) for 48, 72 and 96 h according to the manufacturer’s instructions to establish the optimal transfection time.

LNCaP, DU145 and PC3 cells were treated with 1 µM of the GPER’s agonist G1 (3577, Tocris Bioscience, Bristol, UK) or vehicle (dimethyl sulfoxide, DMSO) for 24 h, at 37 °C in an atmosphere equilibrated with 5% CO_2_. DU145 si-RGN- and si-Scr-transfected cells (24, 48, 72 or 96 h) were also treated with G1 (1 µM) for an additional 24 h.

### 2.4. Real-Time Polymerase Chain Reaction

Total RNA from si-*RGN*- or si-Scr-transfected PNT1A and DU145 cells was isolated using the TripleXtractor reagent (GRiSP, Oporto, Portugal) according to the manufacturer’s instructions. The quantity and integrity of total RNA were determined by measurement of absorbance at 260 and 280 nm (NanoPhotometer; Implen, München, Germany) and agarose gel electrophoresis. cDNA was synthesised from 1 ug of total RNA using the NZY First Strand cDNA Synthesis kit in a 20 µL volume reaction (NZYtech, Lisbon, Portugal), following the manufacturer’s instructions. Quantitative polymerase chain reaction (qPCR) was used to confirm *RGN* gene knockdown. qPCR was carried out in the CFX Connect^TM^ Real-Time PCR Detection System (Bio-Rad, Hercules, CA, USA). The measurements of the efficiency of amplification for all primer sets, as well as all qPCR reactions, were performed as previously described [32,33]. Details about primers, cycling conditions and annealing temperature are depicted in Table 2. *β-2-microglobulin* (*β2M*) was the housekeeping gene used to normalise gene expression, following the Pfaffl mathematical model and using the 2^−(ΔΔCt)^ formula [5].

### 2.5. Cell Viability Assay

PNT1A (5000 cells/well) and DU145 (5000 cells/well) cells were cultured in 96-well plates and, upon 48, 72 and 96 h of si-*RGN* and si-Scr transfection, cell viability was evaluated by the colorimetric 3-(4,5-dimethylthiazol-2-yl)-2,5-diphenyltetrazolium bromide (MTT) assay as described in [65,66]. The number of viable cells is directly proportional to the absorbance (570 nm) measured in each experimental group. The same procedure was used to determine the viability of transfected (si-*RGN* and si-Scr) DU145 cells after treatment with G1 for an additional 24 h.

### 2.6. Fluorescent Immunocytochemistry (Ki-67)

PNT1A (60,000/well) and DU145 (40,000/well) cells were cultured in 24-well plates and, at different experimental time points, Ki-67 immunocytochemistry was performed as reported in [65,66]. In brief, after fixation with 4% paraformaldehyde (PFA), permeabilisation with 1% Triton X-100 and blocking of non-specific staining with phosphate buffer saline containing 0.1% (*w*/*v*) Tween 20 and 20% FBS, cells were incubated with a rabbit anti-Ki-67 (1:50, ab16667, Abcam, Cambridge, UK) primary antibody for 1 h at room temperature. After washing, cells were incubated with Alexa Fluor 546 goat anti-rabbit IgG (1:1000, Invitrogen, Darmstadt, Germany) secondary antibody for 1 h at room temperature and Hoechst 33342 (5 µg/mL, Invitrogen) for 10 min at room temperature. Subsequently, lamellae were washed and mounted using Dako fluorescent mounting medium (Dako, Glostrup, Denmark). The specificity of staining was assessed by omission of the primary antibody. Images were acquired using Zeiss LSM710 laser scanning confocal microscope (Carl Zeiss, Göttingen, Germany). The ratio between Ki-67-positive cells and the total number of cells in 15 randomly selected ×40 magnification fields in each experimental group was calculated as an estimation of a proliferation index.

### 2.7. Protein Extraction

PNT1A, LNCaP, PC3 and DU145 cells were resuspended in radioimmunoprecipitation assay buffer (150 mM NaCl, 1% Nonidet-P40 substitute, 0.5% Na-deoxycholate, 0.1% SDS, 50 mM Tris, 1 mM EDTA) supplemented with 1% protease inhibitor cocktail and 10% phosphatase inhibitor cocktail. Subsequently, cells were lysed as depicted in [33,65,66] and protein concentration was determined using the Pierce™ BCA Protein Assay Kit (Thermo Fisher, Waltham, MA, USA), following the manufacturer’s instructions.

### 2.8. Western Blot

Twenty-five micrograms of total protein extracts was resolved by sodium dodecyl sulfate-polyacrylamide gel electrophoresis on 12.5% acryl-bisacrylamide gel and electro-transferred to polyvinylidene difluoride membranes (Bio-Rad). After blocking with 5% skimmed dried milk for 1 h, membranes were incubated overnight at 4 °C with rabbit anti-RGN (1:2000; ab233007; Abcam). RGN expression was normalised with mouse anti-α-tubulin (1:10,000, T9026). Membranes were incubated for 1 h at room temperature with goat anti-rabbit IgG HRP-linked (1:10,000, #7074, Cell Signaling Technology, Danvers, MA, USA) or anti-mouse IgGκ BP-HRP linked (1:20,000, sc-516102; Santa Cruz Biotechnology) secondary antibodies. Immunoreactive proteins were visualised in a ChemiDoc™ MP Imaging System (Bio-Rad) after incubation with ECL substrate (Bio-Rad) for 5 min. The band density of each sample was quantified using the Image Lab 5.1 software (Bio-Rad) and normalised by division with the respective α-tubulin band density.

### 2.9. Caspase-3-like Activity

Total protein extracts (5 µL) were incubated for 16 h at 37 °C with assay buffer (20 mM HEPES, pH 7.4, 2 mM EDTA, 0.1% CHAPS, 5 mM DTT) and 200 µM of Ac-DEVD-pNA (caspase-3 substrate). Subsequently, the absorbance of the resultant yellow-coloured solution was measured at 405 nm using the xMark™ Microplate Absorbance Spectrophotometer (Bio-Rad). The amount of cleaved caspase-3 substrate produced was assessed by extrapolation using linear regression and normalised to the total quantity (µg) of protein in each experimental condition.

### 2.10. Migration Assay

After transfection with si-*RGN* and si-Scr for 48 h, PNT1A and DU145 cells were collected using 0.25% Trypsin-EDTA solution and resuspended in culture medium. A total of 50,000 cells/chamber were seeded in the upper chamber of the Transwell in the presence or absence of G1 (1 µM). The lower chamber contained culture medium with 10% FBS as a chemoattractant. Cells were maintained at 37 °C and 5% CO_2_ for 24 h. Afterwards, cells on the lower surface of the Transwell were fixed, washed and stained with Hoechst 33342 (5 μg/mL, H3570, Invitrogen). Scanning confocal microscope images (Zeiss LSM710 laser, Carl Zeiss, Göttingen, Germany) were acquired and migration determined by quantifying the total number of Hoechst-stained nuclei in 10 randomly selected ×40 magnification fields per Transwell.

### 2.11. Quantification of Glucose and Lactate

Glucose and lactate contents in PNT1A and DU145 cells’ culture medium were determined spectrophotometrically using commercial kits (glucose: 41010; lactate: 1001330, Spinreact, Girona, Spain) according to the manufacturer’s instructions. Glucose consumption and lactate production were determined relative to the initial concentration of these metabolites at the 0 h time point and normalised to the total quantity (µg) of protein in each experimental condition.

### 2.12. LDH Activity

PNT1A and DU145 total protein extracts were used to determine LDH activity using a commercial kit (41222, Spinreact, Girona, Spain) according to the manufacturer’s guidelines. LDH activity was determined by the rate of NADH consumption in 5 min and normalised to the total amount (μg) of protein in each sample.

### 2.13. Oil Red Assay

PNT1A (60,000/well) and DU145 (40,000/well) cells were stained with Oil Red O as described in [65,66]. Representative staining images were acquired on the Olympus CKX41 inverted phase contrast microscope under ×20 magnification. For quantification of lipid content, Oil Red O was eluted from cells with 100% isopropanol under gentle agitation for 5 min. Absorbance (492 nM) was measured using the xMark™ Microplate Absorbance Spectrophotometer (Bio-Rad).

### 2.14. Statistical Analysis

Statistically significant differences between the experimental groups were evaluated by an unpaired *t*-test with Welch’s correction using GraphPad Prism v8.00 (GraphPad Software, San Diego, CA, USA). Statistical analysis provided by CancerTool included ANOVA, Student *t*-test, Mantel–Cox test and Pearson and Spearman correlations. *p* < 0.05 was considered statistically significant. All experimental data are shown as mean ± S.E.M.

## 3. Results

### 3.1. Downregulated RGN Expression in Metastatic Prostate Cancer Is Related to Poor Survival Outcomes

**Figure 1:**
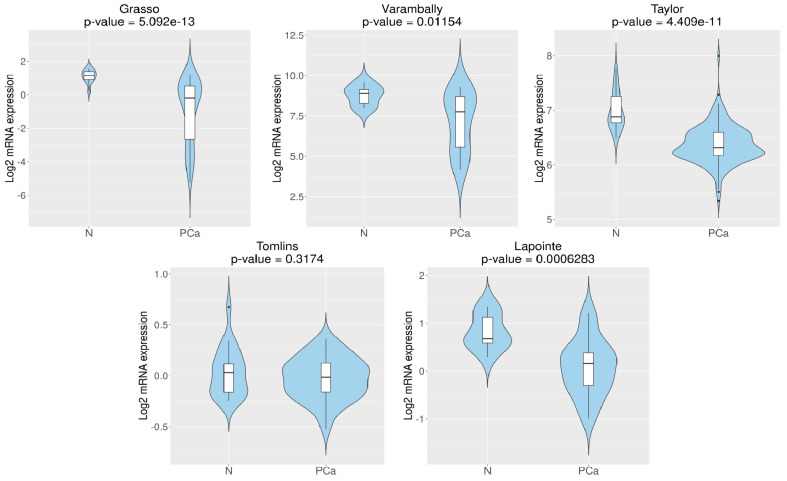
*Regucalcin* (*RGN*) expression in prostate cancer (PCa) and non-PCa (N) patients. *RGN* expression levels were assessed using CancerTool [51]. Violin plots represent *RGN* mRNA expression in N and PCa conditions for the datasets Grasso et al. [53], Varambally et al. [58], Taylor et al. [55], Tomlins et al. [57] and Lapointe et al. [54]. Mean gene expression was compared using a Student *t*-test.

**Figure 2:**
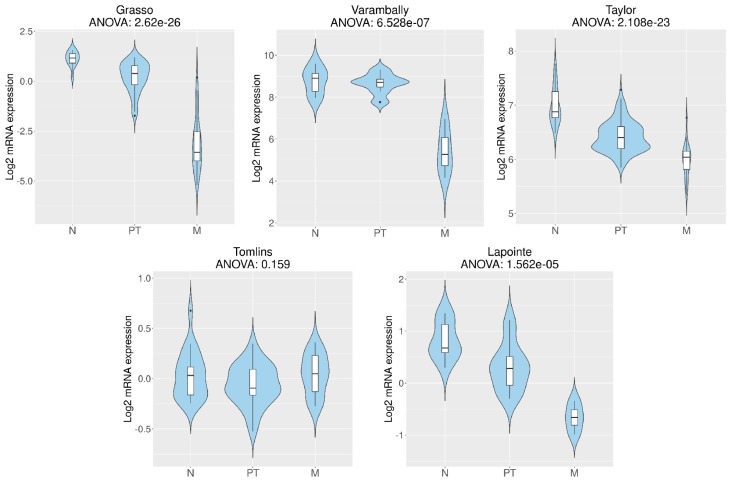
*Regucalcin* (*RGN*) expression with the progression of human prostate cancer (PCa). *RGN* expression levels in non-tumoural prostate (N), primary prostatic tumour (PT) and metastatic PCa (M) were assessed using CancerTool [51]. Violin plots represent *RGN* mRNA expression in N, PT and M for the datasets of Grasso et al. [53], Varambally et al. [58], Taylor et al. [55], Tomlins et al. [57] and Lapointe et al. [54]. Mean gene expression was compared with an ANOVA test.

Using five patients’ datasets (Table 1) and the CancerTool resource [51], *RGN* mRNA expression levels were analysed in non-neoplastic and neoplastic human prostate. *RGN* expression was significantly diminished in PCa cases, considering four (Grasso et al. [53], Varambally et al. [58], Taylor et al. [55] and Lapointe et al. [54]) of the five datasets used (Figure 1). Furthermore, in the Grasso et al. [53], Taylor et al. [55] and Lapointe et al. [54] datasets, *RGN* expression was significantly reduced in metastatic PCa (Figure 2). In the Varambally et al. dataset [58], *RGN* expression did not correlate with the appearance of primary PCa. However, a marked downregulation of *RGN* was found accompanying the progression of the disease to the metastatic stage.

**Figure 3:**
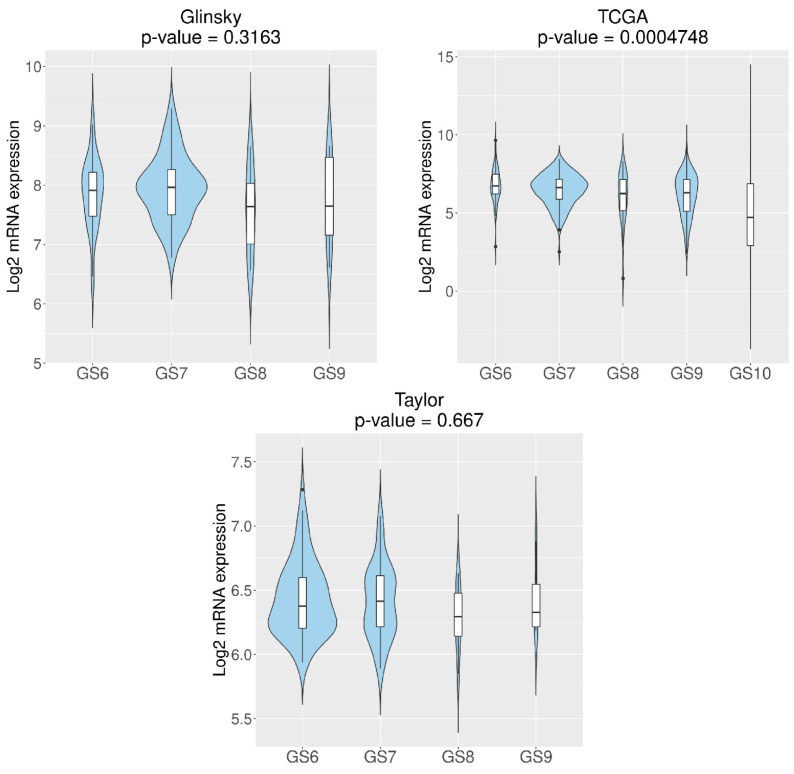
*Regucalcin* (*RGN*) expression according to prostate cancer (PCa) Gleason scores (GS). *RGN* expression levels were assessed using CancerTool [51]. Violin plots represent *RGN* mRNA expression in PCa samples with GS ranging from 6 (GS6) to 10 (GS10) for the datasets of Glinsky et al. [52], TCGA [56] and Taylor et al. [55]. Mean gene expression was compared with an ANOVA test.

**Figure 4:**
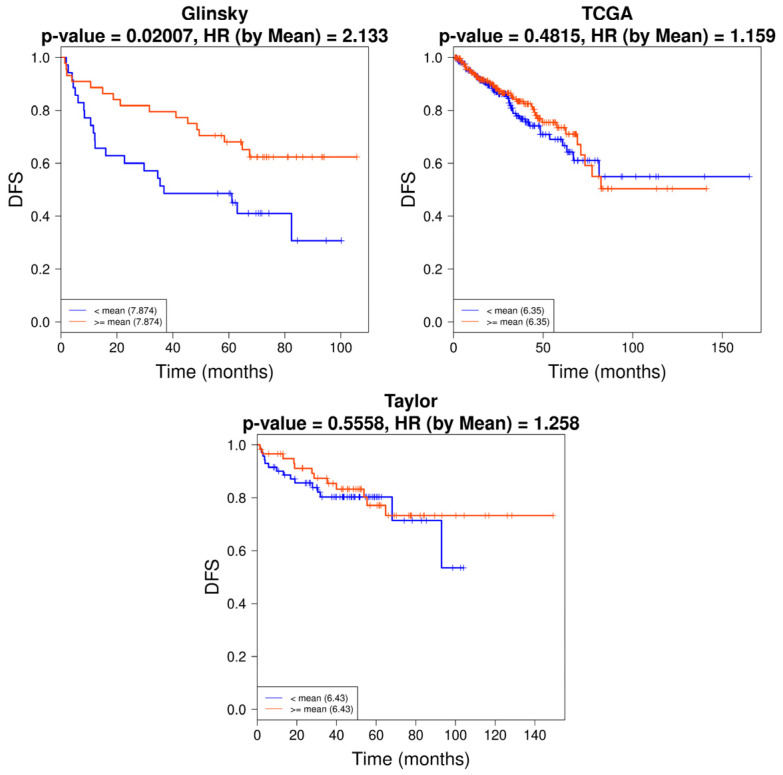
Disease-free survival (DFS) of prostate cancer (PCa) patients according to *regucalcin* (*RGN*) expression levels. *RGN* expression was correlated with DFS using CancerTool [51]. Kaplan–Meier curves represent the DFS of patient groups with high (> or = to the mean, red) or low (<mean, blue) *RGN* mRNA expression levels. Each curve represents the proportion of PCa patients that exhibit DFS over time (months) according to *RGN* expression for the datasets of Glinsky et al. [52], TCGA [56] and Taylor et al. [55]. Censored patients are indicated by the vertical ticks. Groups were compared with a Mantel–Cox test, and a Cox proportional hazards regression model was performed to calculate the hazard ratio (HR).

**Figure 5:**
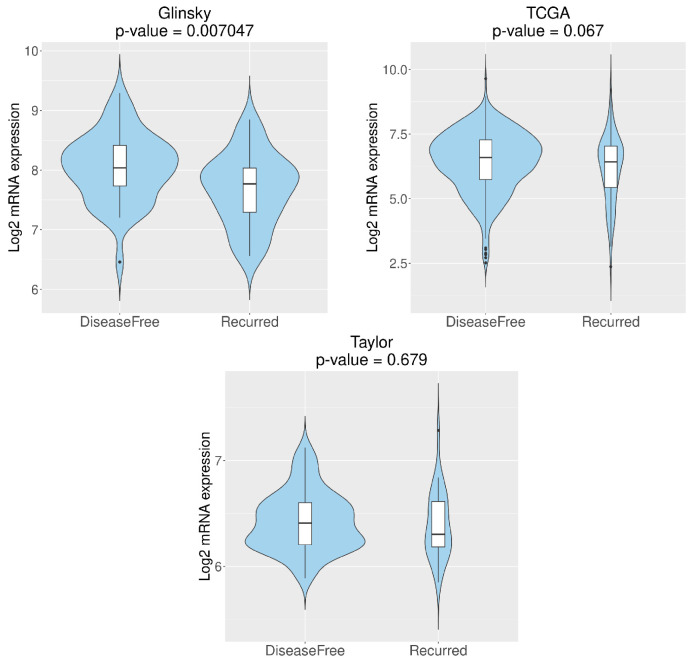
*Regucalcin* (*RGN*) expression in disease-free and recurrent prostate cancer (PCa) patients. *RGN* expression levels were assessed using CancerTool [51]. Violin plots represent *RGN* mRNA expression for the datasets of Glinsky et al. [52], TCGA [56] and Taylor et al. [55]. Mean gene expression was compared with a Student *t*-test.

*RGN* expression levels were also correlated with GS, disease-free survival and recurrence using Glinsky et al. [52], Taylor et al. [55] and TCGA [56] datasets. TCGA [56] dataset demonstrated that loss of *RGN* correlates with GS10 (Figure 3). Moreover, the Glinsky et al. [52] dataset showed that patients with *RGN* expression levels higher than or equal to 2.133 display increased disease-free survival compared to patients with lower *RGN* expression (Figure 4). In alignment, using the same dataset, the analysis confirmed that the decreased expression of *RGN* was directly correlated with PCa recurrence (Figure 5).

### 3.2. RGN Downregulation Differentially Affected PNT1A and DU145 Cell Viability and Proliferation

*RGN* gene expression was silenced in PNT1A (non-neoplastic) and DU145 (neoplastic, androgen non-responsive) human prostate cells by si-*RGN* transfection. qPCR analysis (Supplementary Figure S1) confirmed the downregulation of the *RGN* gene in PNT1A (48 h of transfection) and DU145 (72 h of transfection) cells, with mRNA expression levels reduced by approximately 79% and 27%, respectively.

**Figure 6:**
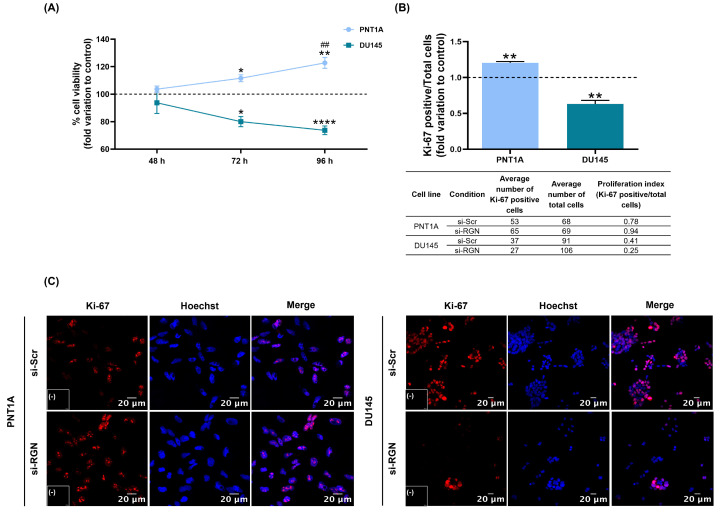
Viability and proliferation of PNT1A and DU145 cells after *regucalcin* (*RGN*) gene knockdown with 10 nM small interfering RNA targeting *RGN* (si-*RGN*) or si-scramble (si-Scr). (**A**) Cell viability assessed by the MTT assay (48, 72 and 96 h of transfection with si-*RGN* relative to the si-Scr group (dashed line). (**B**) Proliferation index after 48 (PNT1A) and 72 h (DU145) of transfection with si-*RGN* relative to the si-Scr group (dashed line). The average numbers of total and Ki-67-positive cells for each cell line and experimental condition are indicated in the table below the graph. (**C**) Representative images of Ki-67-labelled cells (red) and Hoechst-33342-stained nuclei (blue) acquired using the Zeiss LSM 710 laser scanning confocal microscope (400× magnification). Negative controls are shown in the panels (-). Results are expressed as fold change relative to the control group (dashed line; si-Scr). Error bars indicate mean ± S.E.M. * *p* < 0.05; ** *p* < 0.01, **** *p* < 0.0001. ^##^ *p* < 0.01 compared with 72 h.

The effect of *RGN* gene downregulation on PNT1A and DU145 cells’ viability was evaluated by the MTT assay (Figure 6A). si-*RGN*-transfected PNT1A cells presented a significantly augmented viability compared with the si-Scr control group for 72 (112% ± 4.30-fold change) and 96 h (126% ± 6.75-fold change) of transfection. The effect observed was time-dependent, as si-*RGN*-PNT1A cells’ viability at 96 h was significantly higher compared to 72 h (Figure 6A). In DU145 cells, *RGN* downregulation decreased cell viability after 72 (80% ± 6.90-fold change compared to si-Scr group) and 96 h (80 ± 4.97-fold change compared to si-Scr group) of transfection, with no time-dependent effect (Figure 6A).

Ki-67 fluorescent immunocytochemistry was used to assess PNT1A and DU145 cells’ proliferative activity. Upon *RGN* gene knockdown (48 h), an increased number of Ki-67-positive cells was seen in PNT1A cells (1.20 ± 0.05-fold change compared to si-Scr group, Figure 6B). Contrarily, *RGN* knockdown (72 h) reduced the number of Ki-67-positive DU145 cells (0.63 ± 0.10-fold change compared to si-Scr group, Figure 6B). Figure 6C shows representative images of Ki-67 fluorescent immunocytochemistry in PNT1A and DU145 cells.

### 3.3. RGN Gene Downregulation Displayed a Differential Effect on PNT1A and DU145 Cells’ Apoptotic Activity

**Figure 7:**
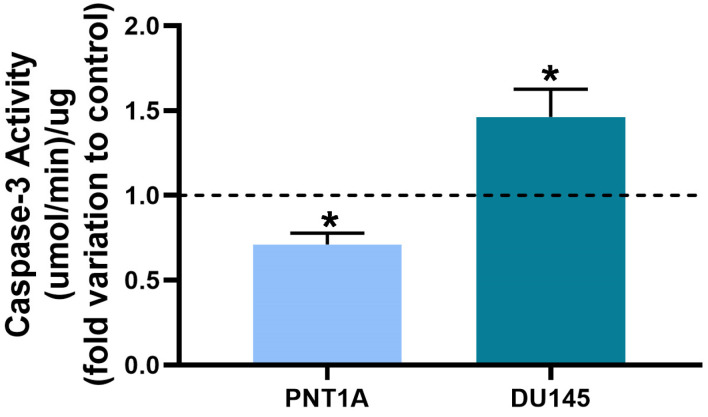
Apoptotic activity of PNT1A and DU145 cells after *regucalcin* (*RGN*) gene knockdown with 10 nM small interfering RNA targeting *RGN* (si-*RGN*) or si-scramble (si-Scr). Caspase-3-like activity was determined by a colorimetric assay. Results are expressed as fold change relative to the control group (dashed line; si-Scr). Error bars indicate mean ± S.E.M. * *p* < 0.05.

Caspase-3-like activity was evaluated in si-*RGN*-transfected PNT1A and DU145 cells. *RGN* gene knockdown significantly increased caspase-3-like activity (1.46 ± 0.20-fold change compared to si-Scr group, Figure 7) in DU145 cells, whereas in PNT1A the opposite effect was observed (0.71 ± 0.09-fold change compared to si-Scr group, Figure 7).

### 3.4. RGN Gene Downregulation Promoted the Migratory Capacity of PNT1A and DU145 Cells

**Figure 8:**
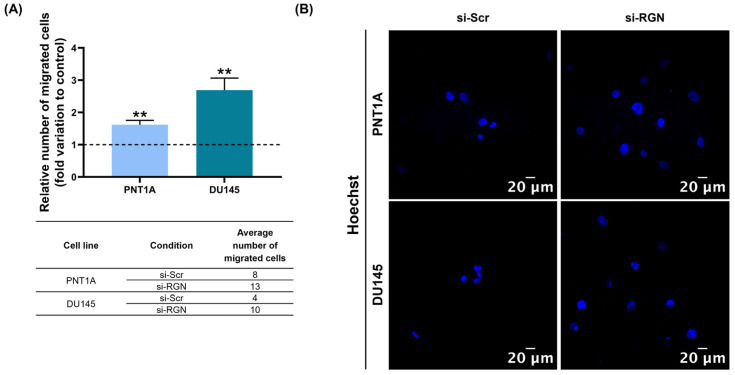
Migratory capacity of PNT1A and DU145 cells after *regucalcin* (*RGN*) gene knockdown with 10 nM small interfering RNA targeting *RGN* (si-*RGN*) or si-scramble (si-Scr). (**A**) Relative number of migrated cells after 72 h transfection determined by a Transwell assay. The average numbers of migrated cells for each cell line and experimental condition are indicated in the table below the graph. (**B**) Representative images of Hoechst-33342-stained nuclei (blue) obtained using the Zeiss LSM 710 laser scanning confocal microscope (400× magnification). Results are expressed as fold change to the control group (dashed line; si-Scr). Error bars indicate mean ± S.E.M. ** *p* < 0.01.

The migratory capacity of si-*RGN*-transfected human prostate cells and controls was assessed through a Transwell assay. After *RGN* knockdown, the migratory capacity of PNT1A and DU145 cells was significantly increased (1.62 ± 0.16 and 2.69 ± 0.48-fold change compared to the si-Scr control, respectively, Figure 8A). Figure 8B shows representative fluorescent images of Hoechst-stained nuclei in PNT1A and DU145 cells upon *RGN* downregulation.

**Figure 9:**
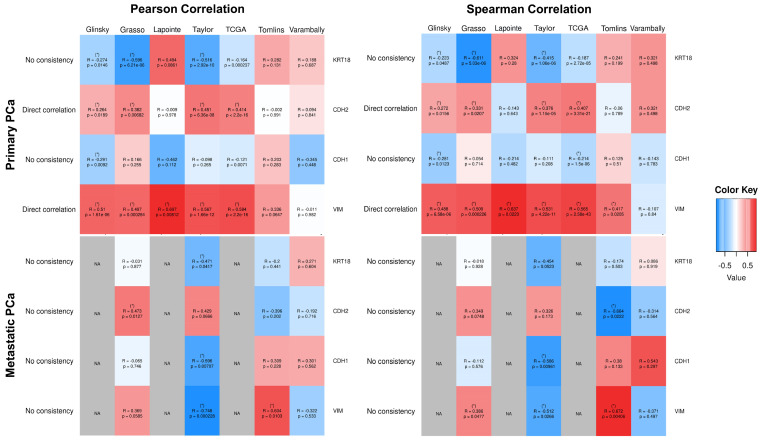
Correlation of *regucalcin* (*RGN*) gene expression with cell migration-associated genes in primary and metastatic prostate cancer (PCa) cases. Heatmaps for Pearson and Spearman correlation analyses of *RGN* gene expression with that of *keratin 18* (*KRT18*), *Cadherin 2* (*CDH2*), *Cadherin 1* (*CDH1*) and *Vimentin* (*VIM*) genes in primary prostate tumours and metastatic PCa were obtained using CancerTool [51] and Glinsky et al. [52], Grasso et al. [53], Lapointe et al. [54], Taylor et al. [55], TCGA [56], Tomlins et al. [57] and Varambally et al. [58] patient datasets. The correlation R-value varied from 1 (direct correlation, red) to −1 (inverse correlation, blue). For datasets containing an insufficient number of samples, the correlation was not applicable (NA, grey cells). (*) indicates correlations with *p* ≤ 0.05 and a correlation coefficient greater than 0.2 (direct) or lower than 0.2 (inverse). Coherence among datasets (more than 50% of datasets with the same correlation pattern) is shown for each pair of genes on the left side.

As a strategy to disclose putative targets of RGN in controlling the migratory capacity of prostate cells, we performed a bioinformatic analysis. The RGN protein network associated with migration and metastasis was evaluated in human PCa cases using the CancerTool resource [51]. Gene-to-gene analyses of *RGN* expression compared with that of keratin 18 (*KRT18*), Cadherin 2 (*CDH2*), Cadherin 1 (*CDH1*) and Vimentin (*VIM*) in primary and metastatic PCa cases were carried out in the Glinsky et al. [52], Grasso et al. [53], Lapointe et al. [54], Taylor et al. [55], TCGA [56], Tomlins et al. [57] and Varambally et al. [58] patient datasets (Figure 9). *RGN* expression correlated with *CDH2* and *VIM* in primary PCa cases; however, no correlation was found in metastatic PCa (Figure 9). Moreover, *RGN* expression did not correlate with *KRT18* and *CDH1* expression in both conditions (Figure 9). The results obtained were consistent for both Pearson and Spearman correlation analyses.

### 3.5. RGN Downregulation Altered the Metabolic Profile of PNT1A and DU145 Cells

**Figure 10:**
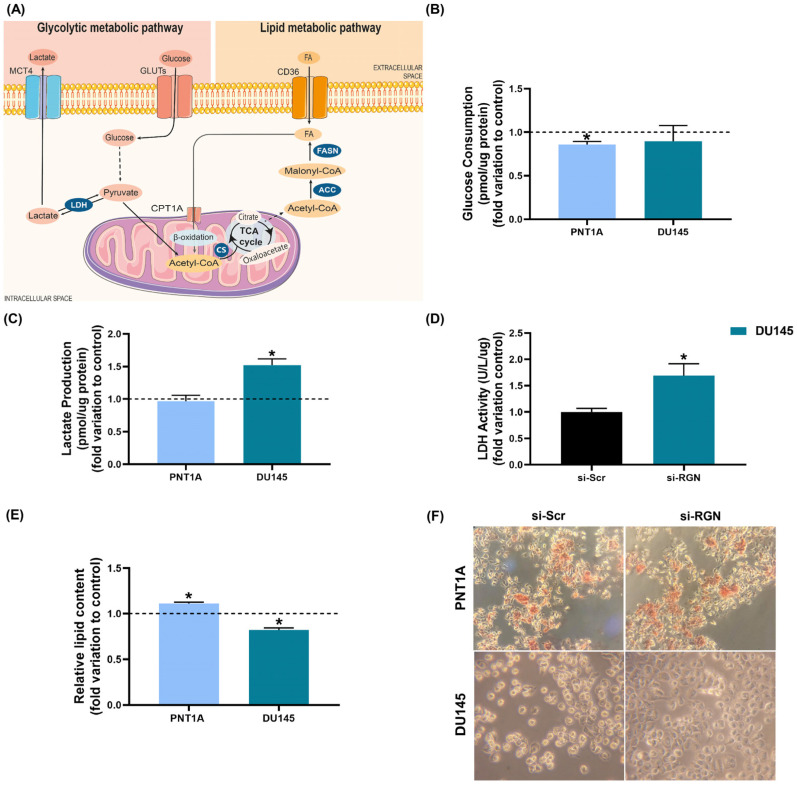
Metabolic profile of PNT1A (48 h) and DU145 (72 h) cells after *regucalcin* (*RGN*) gene knockdown with 10 nM small interfering RNA targeting *RGN* (si-*RGN*) or si-scramble (si-Scr). (**A**) Glycolytic and lipid metabolic pathways. Glucose entering the cell by the activity of glucose transporters (GLUTs) undergoes glycolysis, which culminates in the production of pyruvate. It can be converted to acetyl-coenzyme A (acetyl-CoA) in the mitochondria, entering the tricarboxylic acid (TCA) cycle. In highly glycolytic PCa cells, pyruvate is metabolised by the activity of lactate dehydrogenase (LDH) generating lactate that is exported to the extracellular space through monocarboxylate transporter 4 (MCT4). Fatty acid (FA) uptake from the extracellular space by the CD36 transporter can be directed to storage or oxidation. In the first step of β-oxidation, FA conjugates with carnitine by the activity of carnitine palmitoyltransferase 1A (CPT1A) and is transported to the mitochondria to generate acetyl-CoA that enters the TCA cycle. Next, citrate synthase (CS) catalyses the condensation of acetyl-CoA with oxaloacetate, producing citrate. This intermediary product of the TCA cycle can be exported to the cytosol and converted to acetyl-CoA, which will be used for de novo FA synthesis. The enzymes adenosine triphosphate citrate lyase (ACLY), acetyl-CoA carboxylase (ACC) and FA synthase (FASN) have been shown to be overactivated in PCa cells. Dashed lines indicate that there are intermediate steps not shown. (**B**) Glucose consumption, (**C**) lactate production and (**D**) LDH activity determined spectrophotometrically using commercial kits and normalised to the total quantity (µg) of protein. (**E**) Lipid content assessed by the Oil Red assay. (**F**) Representative images of PNT1A and DU145 Oil-Red-stained cells obtained under 40× magnification. Results are expressed as fold change relative to the control group (dashed line; si-scramble (si-Scr)). Error bars indicate mean ± S.E.M. * *p* < 0.05.

Glycolytic and lipid metabolism (Figure 10A) are energy pathways commonly altered in carcinogenesis, which, in the case of PCa, has been linked to the aggressiveness of disease [65,66,67]. As RGN has been suggested to be implicated in the regulation of glucose and lipid handling [34,35], we decided to investigate whether *RGN* knockdown influences the metabolic profile of human prostate cells.

*RGN* gene knockdown reduced glucose consumption in PNT1A cells (0.86 ± 0.04-fold change compared to si-Scr group, Figure 10B) with no alterations observed in lactate production (Figure 10C). si-*RGN*-transfected DU145 cells presented unaltered glucose consumption (Figure 10B) and increased lactate production (1.52 ± 0.15-fold change compared to the si-Scr group, Figure 10C), which was underpinned by the enhanced LDH activity (1.52 ± 0.13-fold change compared to si-Scr group, Figure 10D).

The effect of *RGN* downregulation in lipid content was evaluated by the Oil Red assay. After *RGN* gene knockdown, lipid content was reduced in DU145 cells (0.82 ± 0.06-fold change compared to si-Scr group, Figure 10E), whereas it was increased in si-*RGN*-transfected PNT1A cells (1.11 ± 0.04-fold change compared to si-Scr group, Figure 10E), as shown in the representative images of Oil-Red-stained PNT1A and DU145 cells (Figure 10F).

### 3.6. RGN Gene Knockdown Enhanced the Anti-Proliferative Effect of GPER in DU145 Cells

**Figure 11:**
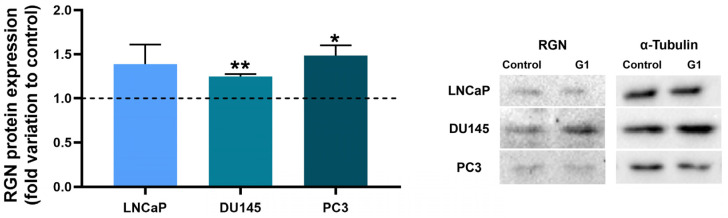
Regucalcin (RGN) expression in LNCaP, DU145 and PC3 cells after treatment with G1 (1 µM) or vehicle (DMSO) for 24 h. Relative protein expression was evaluated by Western blot and normalised with α-tubulin expression. Right panel shows representative immunoblots of three independent assays. Results are expressed as fold change relative to the control group (dashed line). Error bars indicate mean ± S.E.M. * *p* < 0.05; ** *p* < 0.01. The uncropped blots are shown in Figure S2.

RGN protein expression in human LNCaP, DU145 and PC3 PCa cells after 24 h of exposure to GPER agonist G1 or vehicle (DMSO) was assessed by WB analysis. The results in Figure 11 show that RGN expression significantly increased in G1-treated DU145 (1.25 ± 0.04-fold change compared to control group) and PC3 (1.48 ± 0.13-fold change compared to control group) cells. G1 did not significantly affect RGN expression in LNCaP cells.

As GPER regulates RGN expression, and previous findings have indicated that RGN is an oestrogen target gene and that both proteins are associated with the control of cell fate [18,46,48], we investigated the impact of *RGN* gene downregulation on the effects of GPER agonist G1 on cell viability, apoptotic cell death and migratory capacity, using DU145 neoplastic prostate cells as a model.

**Figure 12:**
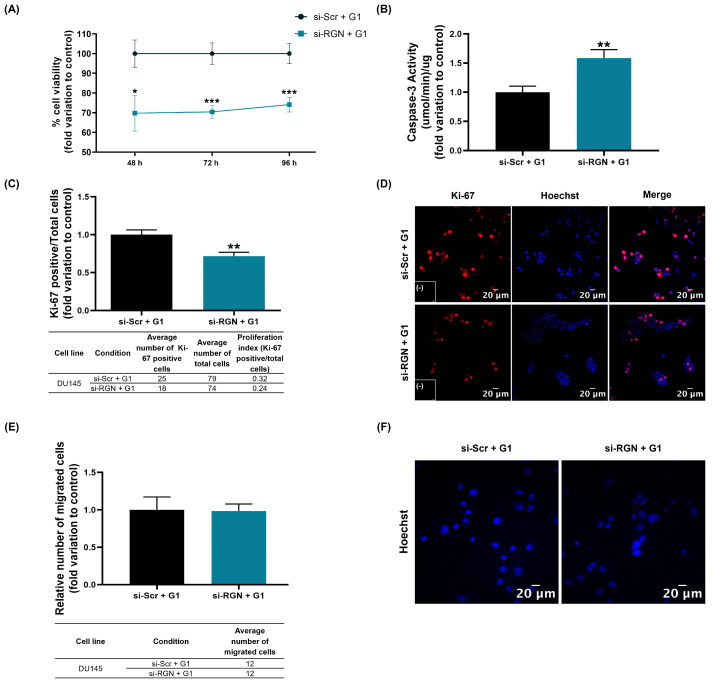
Impact of *regucalcin* (*RGN*) gene knockdown in modulating GPER effects on DU145 cells’ survival and migration. *RGN* gene knockdown was performed with 10 nM small interfering RNA targeting *RGN* (si-*RGN*) or si-scramble (si-Scr) for 48, 72 or 96 h of transfection. Subsequently, cells were treated with the GPER agonist G1 (1 µM) for an additional 24 h (si-Scr + G1 and si-RGN + G1). (**A**) Cell viability assessed by the MTT assay. (**B**) Caspase-3-like activity determined by a colorimetric assay (48 h transfection). (**C**) Proliferation index (48 h transfection). (**D**) Representative images of Ki-67-labelled cells (red), and Hoechst-33342-stained nuclei (blue) acquired using Zeiss LSM 710 laser scanning confocal microscope (400× magnification). Negative controls are shown in the panels (-). (**E**) Relative number of migrated cells determined by a Transwell assay. (**F**) Representative images of Hoechst-33342-stained nuclei (blue) obtained using the Zeiss LSM 710 laser scanning confocal microscope (400× magnification). Results are expressed as fold change relative to the control group (si-Scr + G1). Average numbers of cells, Ki-67-positive cells and migrated cells for each cell line and experimental condition are indicated in the tables below graphs (**C**,**E**). Error bars indicate mean ± S.E.M. * *p* < 0.05; ** *p* < 0.01, *** *p* < 0.001.

Cell viability was assessed in si-*RGN*-transfected DU145 cells for 48, 72 and 96 h followed by 24 h of GPER activation. Cell viability was significantly decreased in G1-treated DU145 cells upon *RGN* knockdown compared to the G1-treated si-Scr-transfected cells regardless of the transfection time (70% ± 11.40, 70% ± 6.29 and 74% ± 3.31 of the si-Scr + G1 control group for 48, 72 and 96 h, respectively, Figure 12A). Concerning the apoptotic cell death response, G1-treated si-*RGN*-transfected DU145 cells displayed an augmented caspase-3-like activity compared to the si-Scr control group (1.59 ± 0.19-fold change, Figure 12B).

GPER activation upon *RGN* gene knockdown significantly decreased the number of Ki-67-positive DU145 cells compared to the si-Scr + G1 control group (0.72 ± 0.08-fold change, Figure 12C). Figure 12D shows representative fluorescent immunocytochemistry images of Ki-67-stained DU145 cells.

The migratory capacity of G1-treated si-*RGN*-transfected DU145 cells was assessed by a Transwell assay. As shown in Figure 12E, no statistically significant differences were observed between G1-treated si-*RGN*-transfected cells (si-RGN + G1) and the control group (si-Scr + G1). Figure 12F shows representative images of Hoechst-stained DU145 cells upon *RGN* downregulation and GPER activation.

### 3.7. RGN Gene Knockdown Together with GPER Activation Enhanced the Glycolytic Profile of DU145 Cells

Available literature has suggested that GPER may play a role in controlling glucose and lipid handling in cancer cells [68]. *RGN* (an oestrogen target gene) has also been linked to the modulation of cell metabolism [34,35,46]. Herein, we investigated the impact of *RGN* gene downregulation on the effects of GPER agonist G1 on glycolytic and lipid metabolism.

**Figure 13:**
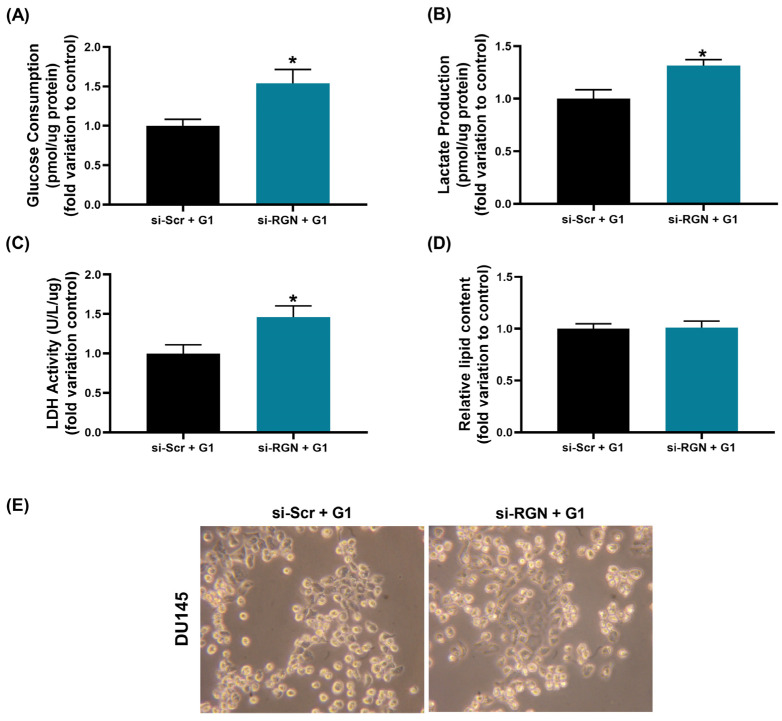
Impact of *regucalcin* (*RGN*) gene knockdown in modulating GPER effects on DU145 cells’ metabolic profile. *RGN* gene knockdown was performed with 10 nM small interfering RNA targeting *RGN* (si-*RGN*) or si-scramble (si-Scr) for 48 h. Subsequently, cells were treated with the GPER agonist G1 (1 µM) for an additional 24 h (si-Scr + G1 and si-RGN + G1). (**A**) Glucose consumption, (**B**) lactate production and (**C**) LDH activity determined spectrophotometrically using commercial kits and normalised to the total quantity (µg) of protein. (**D**) Lipid content assessed by the Oil Red assay. (**E**) Representative images of DU145 Oil-Red-stained cells obtained under 40× magnification. Results are expressed as fold change relative to the control group (si-Scr + G1). Error bars indicate mean ± S.E.M. * *p* < 0.05.

G1-treated si-*RGN*-transfected DU145 cells presented increased glucose consumption (Figure 13A), and lactate production (Figure 13B) compared to the si-Scr + G1 control group (1.54 ± 0.20- and 1.32 ± 0.10-fold change, respectively). Also, LDH activity was augmented in DU145 cells after G1 treatment upon *RGN* gene knockdown (1.46 ± 0.18-fold change compared to si-Scr + G1, Figure 13C). No alterations were observed in the relative lipid content of G1-treated si-*RGN*-transfected DU145 cells determined by the Oil Red assay (Figure 13D) as shown in the representative images of Oil-Red-stained DU145 cells (Figure 13E).

## 4. Discussion

In the present study, we first aimed to investigate how *RGN* knockdown affects human non-neoplastic prostate cell fate and metabolism compared to castration-resistant PCa cells.

*RGN* expression levels in the human PCa datasets were analysed and correlated with patients’ clinicopathological data, PCa progression, GS, disease-free survival and recurrence. CancerTool analysis confirmed the deregulated expression of *RGN* related to the appearance of primary tumours. These findings corroborate the outcomes of our previous report linking the loss of RGN expression with the progression of disease and the cellular differentiation of prostate adenocarcinoma [36]. Moreover, the results obtained herein originally demonstrated that the loss of *RGN* accompanying the progression of disease is significantly related to the evolution of PCa to metastatic stages. Downregulation of *RGN* expression also underpinned poor survival outcomes, i.e., lower disease-free survival and recurrence. It was shown that PCa patients with higher expression levels of RGN display higher disease-free survival and reduced recurrence. With other patient datasets (Gene Expression Omnibus 919 and Gene Expression Omnibus 21034) and cut-offs, other authors have also shown longer PCa recurrence-free and progression-free survival in patients with higher RGN expression levels [37,39]. Altogether, this information suggests that the loss of RGN may be used as a biomarker of PCa aggressiveness and prognosis. Accordingly, TCGA dataset showed that the loss of *RGN* correlates with advanced histological scores (GS10). However, no relationship was found between *RGN* expression levels and GS. Using different datasets to analyse RGN expression levels with the GS, or the variability between datasets, could explain this result. Further studies with larger patient datasets will allow better stratification of patients and establish RGN expression thresholds to increase its usefulness as a prognostic biomarker. Moreover, the correlation of RGN’s prostate immunohistochemistry scores with clinicopathological data, such as PSA levels or GS, will be highly informative and critical for RGN use in clinical settings. As RGN is a secreted protein (see discussion below), the possibility of performing enzyme-linked immunosorbent assays assessing RGN levels in the serum of healthy subjects vs. PCa patients and correlating that with patients’ data is an added value when considering it as a prognostic biomarker.

Notably, the relationship between downregulation of RGN expression and cancer development is not exclusive to PCa. Previous work of our research group showed that breast cancer progression and the advance in the histological grade of infiltrating ductal breast carcinoma are accompanied by the loss of RGN expression [31,36]. The downregulated expression of RGN was also associated with the onset of lung, pancreatic, colorectal, hepatocellular and renal carcinomas [21,22,23,24,30]. These studies have also proposed using RGN to predict patients’ survival and clinical outcomes. In lung squamous cell carcinoma, RGN was also suggested as a potential biomarker of therapy efficacy [69].

Reports on other cancer types and different biological models strongly suggest the utility of using RGN as a prognostic marker. This assumption is highly pertinent, as RGN was identified as a secreted protein in different biological fluids. It was detected in the serum of hepatocellular carcinoma patients in association with neoplastic conditions [70,71]. Interestingly, cDNA microarray analysis in rat liver demonstrated that the diminution of RGN expression appears in pre-neoplastic lesions [72], which supports that loss of RGN precedes the appearance of neoplastic lesions and malignant transformation. This rationale supported the hypothesis that downregulating RGN expression in non-neoplastic prostate cells might establish a pro-tumoural phenotype. Moreover, it is reasonable to assume that decreasing RGN expression levels will alter the proliferative ability and apoptotic response of prostate cells. As indicated by the MTT assay and estimated cell proliferation index assessed by Ki-67 fluorescent immunocytochemistry, *RGN* knockdown increased the viability and proliferative capacity of PNT1A cells which had a time-dependent effect. This is the first report demonstrating that the reduction of RGN expression levels shapes the fate of human non-neoplastic prostate cells, promoting viability and proliferation. However, *in vivo* and *in vitro* studies have widely shown that RGN overexpression suppresses the proliferation of cancer cells. Reports exist on human castration-resistant DU145 and PC3 cells [38,39], rat prostate [13,32,33], liver H4-II-E and kidney NRK52E rat cancer cells [73,74,75] and in human lung, pancreatic, colorectal, hepatocellular, renal, cervical, breast, ovarian and osteosarcoma cell lines [20,21,22,23,24,25,26,27,28]. Therefore, unexpectedly, we observed that *RGN* downregulation increased DU145 cell viability and proliferation. In line with these results, and contrarily to the majority of the available literature, a recent report on MCF-7 and MDA-MB-231 breast cancer cells showed that RGN expression was augmented along with tumour undifferentiation through a mechanism that seems to be mediated by the activation of the ERK/MAPK signalling pathway [76]. As breast cancer and PCa are both hormone-dependent neoplasms, it is viable to assume that the same mechanisms can occur and could trigger the response in the DU145 cell line. The different responses observed between PNT1A and DU145 cells upon *RGN* downregulation highlight that RGN’s effects may depend on its intracellular levels, tumour status or relationship with other molecular targets. Indeed, in other proteins associated with cell survival, namely RNA-binding motif protein 38 and p53, it was observed that, depending on intracellular levels, different cellular responses can be triggered [77].

*RGN* knockdown also differentially affected the apoptotic response of non-neoplastic and neoplastic prostate cells. When *RGN* levels were decreased, PNT1A cells presented a reduced activity of the executioner of apoptosis caspase-3, in line with increased cell viability, whereas the opposite pattern was seen in DU145. As caspase-3 is an end-point of apoptosis, its activity is indicative of the apoptotic rates [78]. Therefore, *RGN* knockdown seems to have an anti-apoptotic effect in PNT1A cells and a pro-apoptotic effect in DU145. This duality of RGN’s actions in the modulation of apoptosis has been described in different tissues and experimental approaches. Our previous study showed that RGN overexpression in rat mammary glands and old rats’ prostates was underpinned by increased caspase-3 activity [13,31]. On the contrary, *in vivo* and *in vitro* experiments also have demonstrated that RGN overexpression suppresses apoptosis [20,21,22,23,25,26,27,30,44,79,80,81,82,83]. However, this inhibition occurred in situations of response to different types of cell damage. The existing evidence indicates that RGN differentially affects the apoptotic response of human prostate cells, depending on its neoplastic condition.

Migration and invasion are the key cellular processes typical of cancer cells, which underlie the development of metastasis, the major cause of cancer-related deaths [84,85]. The obtained results showed that *RGN* knockdown promoted the migratory capacity of PNT1A cells. To the best of our knowledge, this is the first report demonstrating that *RGN* downregulation activates the migratory capacity of non-neoplastic human prostate cells. Moreover, although diminishing viability and proliferative activity, *RGN* knockdown increased the migratory capacity of DU145 cells, which was achieved by suppressing *RGN* expression by only ~27%. Accordingly, other studies have demonstrated that RGN overexpression suppresses cell migration, invasion, angiogenesis and bone metastatic activity in castrate-resistant PCa cell lines [37,38,39,40]. In other cancer types, a relationship was also found between RGN and cell migration and invasion, further indicating that the loss of this protein accelerates tumour aggressiveness and progression towards metastasis [20,21,22,27,30].

**Figure 14:**
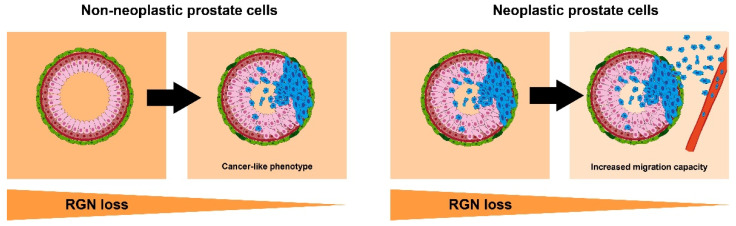
Effect of regucalcin (RGN) loss shaping prostate cell fate. The downregulation of RGN expression in epithelial non-neoplastic prostate cells (pink) induces the development of a highly proliferative and pro-migration cancer-like phenotype. In epithelial neoplastic cells (blue), loss of RGN increases their migration activity and metastatic behaviour, augmenting prostate cancer aggressiveness. Basal and stroma prostate cells are shown in red and green, respectively.

Considering the survival, proliferation and migration results, it is noteworthy that the knockdown of *RGN* expression levels increased PNT1A cell viability, proliferation and migration activity, whereas DU145 cell migration was increased and viability and proliferation were decreased. These results allow the assumption that the loss of RGN in non-neoplastic prostate cells promotes a shift to a cancer-like phenotype, i.e., highly proliferative and pro-migration, whereas in neoplastic cells it could drive metastasis as a consequence of increased migration capacity (Figure 14). Despite these assumptions, the molecular mechanisms that are disrupted by the absence of RGN and responsible for driving the alterations in prostate cell fate need to be disclosed. Nevertheless, the obtained findings partially explain why disease-free survival is increased and recurrence reduced in PCa patients with higher RGN expression levels, as it could mean slower tumor development and lower metastatic potential. The same relationship, i.e., high RGN and high survival rates, has been reported in lung, pancreatic, colorectal, hepatocellular and renal carcinomas [21,22,23,24,30].

We can also discuss that the decreased proliferative rate of DU145 cells in response to *RGN* gene knockdown may represent an adaptative change that allows increased migration, which in an *in vivo* scenario may represent enhanced survival in new environmental conditions and increased metastatic capacity. A study on breast cancer cells, showing that upon entering epithelial–mesenchymal transition, cells lower proliferative activity to survive in hostile new environments [86], supports this hypothesis.

To have an insight into the protein network underlying RGN’s effects on the modulation of cell migration, we used bioinformatic analysis to analyse its expression in correlation with that of *KRT18*, *CDH2*, *CDH1* and *VIM* in primary and metastatic PCa cases. Bioinformatics analysis using several PCa patients’ datasets (CancerTool) demonstrated that *RGN* expression directly correlates with *CDH2* and *VIM* gene expression in primary PCa cases. In a study with human cervical adenocarcinoma HeLa cells, it was reported that *RGN* knockdown increased migration, invasion and metastisation through the upregulation of N-cadherin and vimentin [27]. The “cadherin switch”, characterised by the upregulation of N-cadherin and downregulation of E-cadherin, together with the upregulation of intermediated filament proteins, such as vimentin, is intimately connected with increased cell migration and invasion and by consequence augmented cancer aggressiveness [87,88]. Therefore, these bioinformatic results of *RGN* expression correlated with N-cadherin and vimentin together with the increased migratory capacity of DU145 upon *RGN* knockdown support patients’ data associating loss of RGN with metastisation.

Typically, cancer cells undergo a metabolic switch preferentially using glycolysis as the primary ATP source. They rely on enhanced glucose consumption and high lactate production, even in aerobic conditions, which has been shown to sustain the intense cell proliferation activity and tumour growth [67]. Nonetheless, PCa exhibits unique metabolic features [67]. In the early stages, PCa cells present increased rates of oxidative phosphorylation and use lipid metabolism [67]. Only at advanced stages of the disease does oxidative phosphorylation give way to the hyperglycolytic profile [67]. The liaison between RGN and cell metabolism has been studied, and its role in regulating glycolytic and lipid metabolism has been demonstrated [89]. We previously showed that glucose and lactate concentrations and LDH activity are significantly reduced in the prostate of transgenic rats overexpressing RGN [33]. In line with these findings, herein we found that *RGN* downregulation increased the glycolytic metabolism of PCa DU145 cells, as indicated by the increased lactate production, underpinned by an increased LDH activity. To the best of our knowledge, this is the first report of RGN’s actions in the modulation of cancer cell metabolism and targeting this relevant cancer hallmark.

Concerning lipid metabolism, a differential response was seen after *RGN* knockdown in PNT1A and DU145 cells. Lipid content was increased in PNT1A cells by reducing *RGN* expression levels and decreased in DU145. This suggests that loss of RGN expression, besides increasing the glycolytic profile of DU145 cells, may recruit lipids from storage, which could sustain the observed increase in their migratory capacity. In fact, it has been described that PCa cells sustain metastatic behaviour by increasing fatty acid oxidation, glycolysis and lactate production [90,91]. Further studies are needed to clarify how RGN works in lipid handling in PCa cells. However, its relationship with lipid metabolism was demonstrated in the liver of aged rats. RGN overexpression increased triglyceride, total cholesterol, free fatty acid and neutral lipid contents [29,34,35].

The research efforts to unveil the mechanisms that govern RGN expression have identified it as an oestrogen target gene [46,47,92]. Moreover, 17β-oestradiol stimulation experiments in MCF-7 cells suggested the involvement of the GPER [36] in the regulation of *RGN* gene transcription, which led us to hypothesise that the reported GPER anti-tumourigenic actions can partially result from the modulation of RGN expression levels. WB analysis confirmed that GPER activation by its agonist G1 increased RGN expression in the castration-resistant PCa cells.

To identify the existence of an GPER/RGN interplay, the *RGN* gene was knocked down in DU145 cells and their response to the GPER agonist G1, concerning different cancer hallmarks, was assessed. DU145 cells were used as a model for addressing this aim as they mimic metastatic stages of advanced PCa [62,63], which could generate more meaningful information for clinical settings because of the relationship of RGN with the metastatic process, prognosis and disease-free survival. It was observed that upon suppression of *RGN* expression levels, G1/GPER decreased the viability and proliferative rate of DU145 cells. Also, it increased the apoptotic rate, as indicated by the augmented activity of caspase-3, though no alterations were found in cell migration level. Concerning glycolytic and lipid metabolism, we observed that *RGN* knockdown and G1 treatment increased glucose consumption, lactate production and LDH activity in DU145 cells, but no alterations were perceived in lipid content. These results resemble those of *RGN*-knockdown DU145 cells, indicating that the loss of RGN superseded the expected G1/GPER actions positively regulating RGN expression levels.

Although these results are preliminary and no comparable findings have been reported, the present study corroborates the role of GPER in regulating RGN expression. Also, they highlight the existence of a GPER/RGN interplay in modulating the behaviour of metastatic PCa cells. Further studies should determine if the relationship exists and also has an impact on non-neoplastic cells. Nevertheless, the obtained findings open new avenues of possibilities in cancer research, namely in understanding the usefulness of these molecular targets in the management and treatment of PCa.

## 5. Conclusions

This study first demonstrated that loss of RGN induces the development of cancer-like characteristics in non-neoplastic prostate cells and augments the aggressiveness of castration-resistant PCa cells. Moreover, these *in vitro* findings aligned with patients’ data, where lower *RGN* expression was correlated with the development of metastatic PCa and poor survival outcomes. It was also confirmed that GPER activation increases RGN expression levels in castration-resistant PCa cells and that RGN expression levels may influence GPER action. However, the molecular mediators of this relationship remain to be unveiled and identifying the interplay between GPER and RGN will open new avenues of research. Disclosing their mechanisms of action will be pivotal in exploring their potential as biomarkers or therapeutic targets in PCa.

## Figures and Tables

**Table 1:** Details of patients’ datasets used in the in silico bioinformatic analysis.

Dataset	GEO Accession Number	Total Number of Patients	Number of Patients Per Disease Status				Reference
			Non-Tumoural	Primary Tumour	Metastatic Cancer	Other	
Glinsky et al.	Donated by Memorial Sloan Kettering Cancer Center	79	-	79	-	-	[52]
Grasso et al.	GSE35988	88	12	49	27	-	[53]
Lapointe et al.	GSE3933	26	9	13	4	-	[54]
Taylor et al.	GSE21032	183	29	131	19	4	[55]
TCGA	-	497	-	497	-	-	[56]
Tomlins et al.	GSE6099	104	23	32	20	29	[57]
Varambally et al.	GSE3325	19	6	7	6	-	[58]

**Table 2:** Primers and real-time polymerase chain reaction cycling parameters.

Gene/GenBank Accession Number	Primer Sequence (5′–3′)	Primer Concentration (nM)	Amplicon Size (bp)	AT (°C)	Number of Amplification Cycles
*Regucalcin*/AB032064	Sense: GCAAGTACAGCGAGTGACC Antisense: TTCCCATCATTGAAGCGATTG	300	177	60	40
*β-2-microglobulin*/NM_004048.2	Sense: ATGAGTATGCCTGCCGTGTG Antisense: CAAACCTCCATGATGCTGCTTAC	300	93	60	40

AT: Annealing temperature.

## Data Availability

The data presented in this study are available in this article.

## Supplementary Materials

The following supporting information can be downloaded at: https://www.mdpi.com/article/10.3390/cancers16233932/s1, Figure S1: Regucalcin (RGN) mRNA expression levels in PNT1A (48 h) and DU145 (72 h) cells after transfection with the small interfering RNA targeting RGN or si-scramble; Figure S2: Original Western blots images after regucalcin and α-tubulin immunodetection in (A) LNCaP, (B) DU145 and (C) PC3 cells protein extracts after treatment with G1 (1 µM) or vehicle (DMSO) for 24 h.
