# Supplementary figures and images for: Downregulated Regucalcin Expression Induces a Cancer-like Phenotype in Non-Neoplastic Prostate Cells and Augments the Aggressiveness of Prostate Cancer Cells: Interplay with the G Protein-Coupled Oestrogen Receptor?
Source: Cancers (Basel). 2024 Nov 24;16(23):3932. doi: 10.3390/cancers16233932 (PMC11640704; doi:10.3390/cancers16233932)

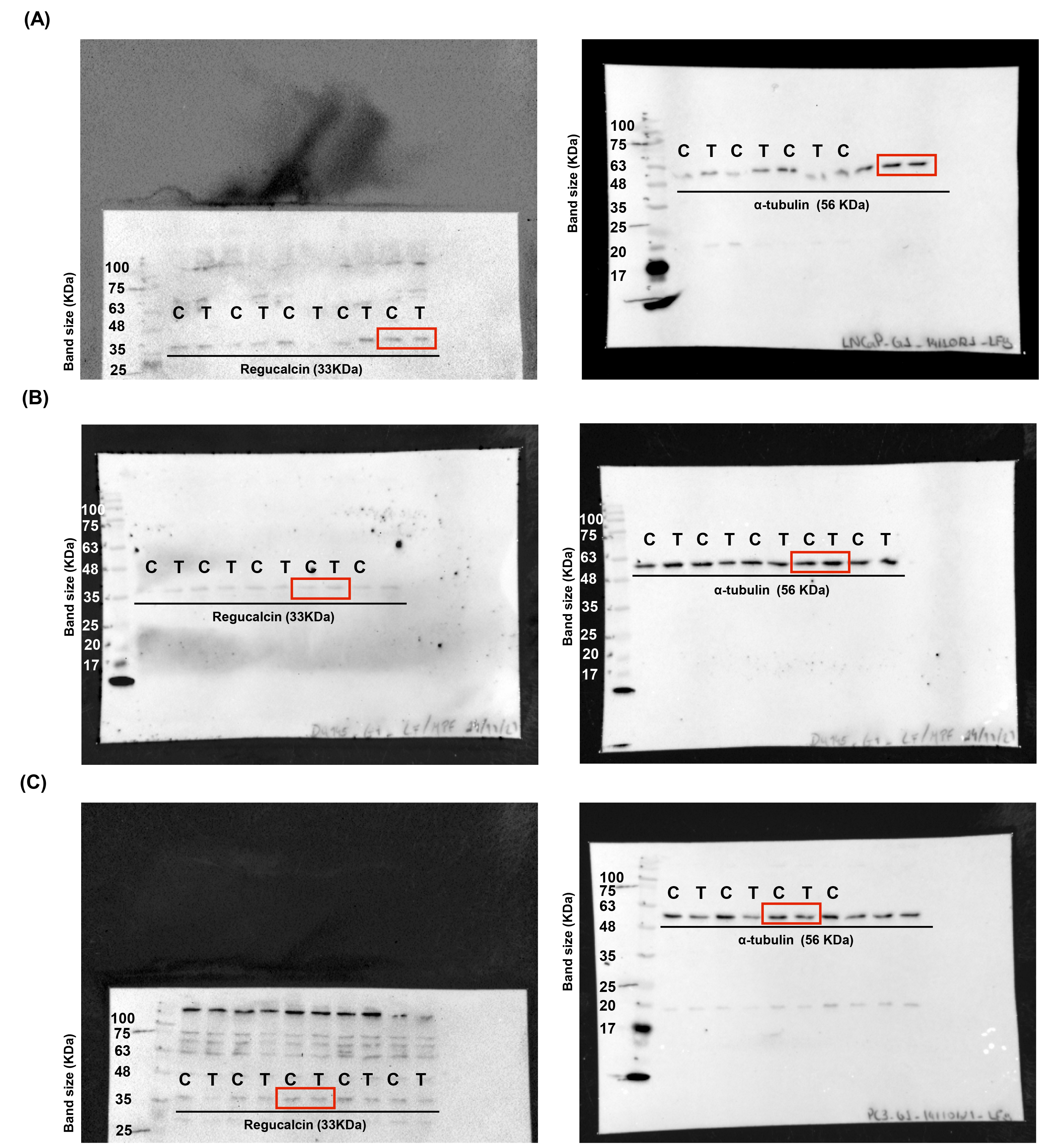

Supplement: Supplementary file 1 [file cancers-16-03932-s001.zip › Figure S2.tif]
